# Sulforaphane-Enriched Extracts from Broccoli Exhibit Antimicrobial Activity against Plant Pathogens, Promising a Natural Antimicrobial Agent for Crop Protection

**DOI:** 10.3390/biom14030352

**Published:** 2024-03-14

**Authors:** Lixia He, Hanmin Jiang, Yaotong Li, Xu Zhang, Wenting Sun, Ce Liu, Zekai Zhao, Chengrong Yun, Hui Li, Chunguo Wang

**Affiliations:** 1 College of Life Sciences, Nankai University, Tianjin 300071, China; helx@mail.nankai.edu.cn (L.H.); 2120211178@mail.nankai.edu.cn (Y.L.); 1120220631@mail.nankai.edu.cn (X.Z.); 1120230739@mail.nankai.edu.cn (W.S.); 98020220004@nanaki.edu.cn (C.L.); 2120211268@mail.nankai.edu.cn (Z.Z.); 2120221394@mail.nankai.edu.cn (C.Y.); 2 Tianjin Academy of Agricultural Sciences, Tianjin 300380, China; 2120221501@mail.nankai.edu.cn; 3 College of Horticulture and Landscape, Tianjin Agricultural University, Tianjin 300384, China; lihui@tjau.edu.cn

**Keywords:** broccoli, SFN-enriched extracts, antimicrobial activity, ROS accumulation, crop protection

## Abstract

Sulforaphane (SFN) is one of the hydrolysates of glucosinolates (GSLs), primarily derived from *Brassica* vegetables like broccoli. In clinical therapy, SFN has been proven to display antimicrobial, anticancer, antioxidant, and anti-inflammatory properties. However, the antimicrobial effects and mechanism of SFN against plant pathogens need to be further elucidated, which limits its application in agriculture. In this study, the genetic factors involved in SFN biosynthesis in 33 *B. oleracea* varieties were explored. The finding showed that besides the genetic background of different *B. oleracea* varieties, *myrosinase* and *ESP* genes play important roles in affecting SFN content. Subsequently, the molecular identification cards of these 33 *B. oleracea* varieties were constructed to rapidly assess their SFN biosynthetic ability. Furthermore, an optimized protocol for SFN extraction using low-cost broccoli curds was established, yielding SFN-enriched extracts (SFN-ee) containing up to 628.44 μg/g DW of SFN. The antimicrobial activity assay confirmed that SFN-ee obtained here remarkably inhibit the proliferation of nine tested microorganisms including four plant pathogens by destroying their membrane integrity. Additionally, the data demonstrated that exogenous application of SFN-ee could also induce ROS accumulation in broccoli leaves. These results indicated that SFN-ee should play a dual role in defense against plant pathogens by directly killing pathogenic cells and activating the ROS signaling pathway. These findings provide new evidence for the antimicrobial effect and mechanism of SFN against plant pathogens, and suggest that SFN-ee can be used as a natural plant antimicrobial agent for crop protection and food preservation.

## 1. Introduction

Plants are exposed to a wide variety of pathogens in natural ecosystems, which can reduce crop yields and cause substantial economic losses. The major global crop losses caused by pathogens and pests range from 17% to 30% [1]. Therefore, the prevention and treatment of plant diseases are crucial for plant survival and productivity, as well as the global economy. Over the past decades, chemical fungicides have been the main prevention strategy for pathogen control [2]. However, various agricultural pesticides and other fungicides have persistent, bioaccumulation properties and are highly toxic [3]. Long-term use of these chemical fungicides can result in pathogen resistance, environmental contamination, and noxious residue accumulation in food, which seriously endangers crop production and human health. Consequently, more eco-friendly and safer alternatives for pathogen control are in huge demand in modern agriculture [2]. Natural antimicrobial substances, especially plant extracts, are considered safe, low in toxicity, and highly decomposable, raising particular focus on their application in plant disease control [4,5]. Plant extracts containing bioactive components are also endorsed in the food industry as natural, eco-friendly, safe, and easily biodegradable agents for food preservation [6,7]. Nevertheless, more natural substances from diverse plants displaying strong antibacterial activity are still required to be explored and identified for the development of novel biocontrol agents.

Sulforaphane is one of the most important bioactive compounds in cruciferous vegetables, such as broccoli and cauliflower [8], and is derived from glucosinolates (GSLs), which are the nitrogen- and sulfur-containing plant secondary metabolites. GSLs are hydrolyzed by myrosinases to produce SFN [9]. However, SFN is not a unique hydrolysate of GSLs. Another enzyme, named epithiospecifier protein (ESP), hydrolyzes GSLs to produce undesirable nitrile components, resulting in a low SFN yield [10]. SFN has been well known for its anticarcinogenic, antioxidant, and anti-inflammatory effects in humans [11]. Moreover, SFN has shown effective antimicrobial potency against human pathogens. For instance, SFN inhibits *S. dysenteriae* by negatively regulating the expression of Shiga toxin [12]. SFN and related compounds exhibit a strong inhibitory effect on *H. pylori*, a bacterium responsible for human gastritis and stomach cancer, and effectively impair the virulence of *V. cholerae* [13,14,15]. Available evidence in the model plant *A. thaliana* suggested that SFN also has antimicrobial potential against plant pathogens. In *Arabidopsis*, SFN is confirmed to inhibit *P. syringae* by primarily regulating the type III secretion system (TTSS) genes, which are essential for pathogenesis. SFN-deficient plants exhibit increased susceptibility to *P. syringae* strains [16]. Similarly, SFN can activate defense priming and reduce susceptibility to *H. arabidopsidis* infection in *Arabidopsis*, thereby reducing sensitivity to downy mildew [17]. Obviously, SFN, as a natural plant compound, is expected to be a promising candidate for novel harmless plant and food protective agents. However, the antibacterial activity and mechanism of SFN in defending various plant pathogens in crops are still unclear. The development and utilization of SFN as biocontrol agents have also been limited.

In the present study, the genetic background of 33 *B. oleracea* varieties was investigated by simple sequence repeats (SSRs) analysis and the molecular identification (ID) of each variety was then established. Furthermore, the correlation of GSL and SFN content with the genetic background and the expression levels of *myrosinase* and *ESP* in broccoli cultivars was evaluated. The broccoli cultivars with high SFN content were identified and utilized as materials to quantitatively extract SFN using an optimized method. Subsequently, the antimicrobial activity of the SFN-ee was verified. The antimicrobial mechanism of the SFN-ee was explored and further discussed.

## 2. Materials and Methods

### 2.1. Plant Materials and Microorganism Strains

A total of 33 *B. oleracea* varieties including 29 broccoli cultivars and 4 cauliflower cultivars were collected (Table S1). The seeds of the 33 varieties were grown in a greenhouse with a temperature regime of 25/23 °C, 16/8 h day/night cycle, and a relative humidity ranged from 55% to 65%. The 7-day-old seedlings were harvested to determine GSLs and SFN contents. In addition, four bacterial strains including *X. campestris* (CGMCC 1.1781), *S. aureus* (CGMCC 1.282), *E. coli* (CGMCC 1.12883), and *A. rhizogenes* (ATCC 15834), and five fungal strains including *F. oxysporum* (ATCC 48112), *G. zeae* (ATCC 60309), *P. infestans* (ATCC 52167), *C. neoformans* (ATCC 90113), and *P. pastoris* (CGMCC 23705), were used to evaluate the antimicrobial activity of SFN-ee. *X. campestris* was cultured in Trypticase Glucose Yeast (TPGY) extract broth. *S. aureus*, *E. coli*, and *A. rhizogenes* were cultured in Luria-Bertani (LB) medium. *F. oxysporum*, *G. zeae*, and *P. infestans* were cultured in Potato Dextrose Agar (PDA) medium. *P. pastoris* and *C. neoformans* were cultured in Yeast Extract Peptone Dextrose Adenine (YPDA) medium. All these bacterial and fungal strains are stored at the College of Life Sciences, Nankai University, Tianjin, China.

### 2.2. DNA Preparation and Genetic Diversity Analysis by SSR Method

Total genomic DNAs were extracted from the 7-day-old seedlings of 33 *B. oleracea* varieties using the CTAB method with modifications [18]. The quality and quantity of the DNAs were assessed using 1% agarose gels and a Nanodrop 1000 (Nanodrop, Wilmington, DE, USA). Brassica SSR primer pairs were obtained from previous papers [19,20,21,22], and a total of 39 SSR primer pairs were prescreened for polymorphism among 33 distinct commercial varieties (Table S2). An improved SSR method was conducted as described by Zhu et al. [20]. In brief, the PCR reaction in a 25 μL volume containing DNAs (50 ng), MgCl_2_ (2.0 mM, Cat:R0058, Beyotime, Shanghai, China), dNTPs (0.2 mM, Cat#4019, Takara, Dalian, China), SSR primer pairs (0.5 μM, Sangon, Beijing, China), and Taq DNA polymerase (0.5 U, Cat#R004, Takara, Dalian, China) was performed in Bio-Rad MyCycler Thermal Cycler System (Bio-Rad, Santa Clara, CA, USA). The procedure was as follows: an initial denaturation step for 3 min at 94 °C, 15 s at 94 °C, 15 s at the annealing temperature depending on the primers, and 1 min at 72 °C for 30 cycles, followed by an extension step of 5 min at 72 °C. Then, the reaction was maintained at 10 °C. The SSR primer-amplified products from the 33 samples were separated by electrophoresis on 8.0% non-denatured polyacrylamide gels (PAGE) for 2 h at 180 V. The DNA polymorphic fragments on the gels were visualized by rapid silver staining. Finally, Nei’s genetic distance matrix data were calculated for all samples on the basis of the SSR markers, which was performed to construct a dendrogram based on the neighbor-joining (NJ) method in MEGA 6.0 software [23].

### 2.3. Determination of Total GSLs

The content of total GSLs was determined by a modified palladium chloride colorimetry method [24]. In brief, fresh 7-day-old seedlings of the 33 *B. oleracea* varieties were taken and dried in an oven at 110 °C for 1 h. Next, 0.1 g of the dried material was frozen in liquid nitrogen and then ground to powder in a mortar and pestle, at room temperature. The powders were hydrolyzed in a total volume of 10 mL of hot water (100 °C). The mixture, including 1 mL filtrated hydrolysate, 2 mL 0.15% sodium carboxymethylcellulose (CAS:9004-32-4, Macklin, Shanghai, China), and 1 mL palladium chloride (CAS:7647-10-1, Aladdin, Shanghai, China), reacted at 25 °C for 3 h. Then, the absorbance values were measured at 540 nm, using palladium chloride and sodium carboxymethylcellulose solution as references. The content of total GSLs was determined by the standard curve method.

### 2.4. SFN Extraction and HPLC Assay

The modified SFN extraction methods were conducted as described by Matusheski et al. [25]. In brief, fresh broccoli curds or 7-day-old seedlings were dried at 40 °C to constant weight. The dried raw materials with a constant weight of 1 g were frozen in liquid nitrogen and crushed into powders in a mortar and pestle, at room temperature. They were then hydrolyzed with phosphate buffer (pH 6.0, BN20326, Biorigin, Beijing, China) for 12 h at 50 °C. The hydrolysates were extracted three times with dichloromethane (CAS:75-09-2, Guangshunda, Tianjin, China). All extracts were combined and 2 g of Na_2_SO_4_ (CAS:7757-82-6, Guangshunda, Tianjin, China) added to absorb water, followed by sonication at 80 Hz for 60 min. Finally, the dichloromethane extractants were evaporated at 45 °C using a Rotary Evaporator RE-52AA (Yarong, Shanghai, China). The SFN-ee were dissolved in methanol (CAS:67-56-1, Guangshunda, Tianjin, China) for further HPLC–UV (Elite, Dalian, China) analysis at 235 nm. A 48 min gradient program was used in the HPLC–UV system. Briefly, acetonitrile (CAS:75-05-8, Guangshunda, Tianjin, China) was eluted at 20–60%, and ultrapure water containing 0.1% phosphoric acid (CAS:7664-38-2, Guangshunda, Tianjin, China) was eluted at 80%–40% for 0–20 min. Acetonitrile gradually increased up to 80% within 20–25 min, then gradually returned to 20% acetonitrile over 3 min and held at 20% acetonitrile for 7 min. SFN (≥95%; CAS:4478-93-7, Sigma-Aldrich, Shanghai, China) was used as a standard and conducted to serial dilutions to construct the standard curve.

### 2.5. RNA Isolation and Quantitative Real-Time RT-PCR (qRT-PCR) Analysis

Total RNAs were isolated from the 7-day-old seedlings with TRIzol™ Reagent according to the manufacturer’s instructions (Invitrogen, Carlsbad, CA, USA). The quality and quantity of the RNAs were assessed by 1% agarose gels and a Nanodrop 1000 (Nanodrop, Wilmington, DE, USA). First-strand cDNA synthesis was performed using 1 μg of RNAs in a total volume of 20 μL according to HiScript^®^ II 1st Strand cDNA Synthesis Kit (Vazyme, Nanjing, China) instructions. The transcriptional expression levels of representative genes such as *myrosinase* and *ESP* were validated by qRT-PCR on a BIORAD IQ^TM5^ Fast Real-Time PCR System (Bio-Rad, Santa Clara, CA, USA). The specific primer pairs designed by Primer premier 5.0 software (Table S3) and FastStart Universal SYBR Green Master Mix (Roche, Basel, Switzerland) were used in the qRT-PCR assay. The data were presented after normalization to the reference gene *BolActin* from broccoli via the comparative 2^−ΔΔCT^ method (Table S3). Three biological replicates and three technical replicates were performed to ensure the reliability of the quantitative data.

### 2.6. Detection of Antimicrobial Activity of SFN-ee In Vitro

The antibacterial activity of SFN-ee was detected according to the Kirby–Bauer disk diffusion method with modifications [26]. Briefly, 100 μL bacteria or fungus solution with the final concentration of 10^8^ CFU/mL was evenly spread on the surface of the corresponding solid medium. The disks loading 20 μL SFN-ee, which contained 10 or 20 μg SFN, were then placed on the petri dishes with different microbial strains. Methanol was used as the negative control and sterile water was used as the blank control. After 18–48 h incubation at 37 °C for bacterial strains and 30 °C for fungal strains, the inhibitory activity of SFN-ee was evaluated by measuring the diameter of inhibition zone. In addition, to evaluate the antimicrobial activity of SFN-ee, statistical analysis was conducted on the diameters of the inhibition zones produced by SFN-ee and five reported representative antimicrobial agents against plant pathogens under two dose levels (10 or 20 μg). These five antimicrobial agents were nisin (CAS 1414-45-5, water-soluble, Ekear, Shanghai, China), cefotaxime sodium (CAS 64485-93-4, water-soluble, Yuanye, Shanghai, China), chitosan (CAS 9012-76-4, dissolved in 1% acetic acid, Macklin, Shanghai, China), potassium sorbate (CAS 24634-61-5, water-soluble, Macklin, Shanghai, China), and catechin (CAS 154-23-4, water-soluble, Macklin, Shanghai, China), and they were used as positive controls. The Kirby–Bauer disk diffusion method was the same as above.

### 2.7. Determination of the Minimum Inhibitory Concentration (MIC)

The MIC of SFN-ee was determined by two-fold serial dilution method [27]. In brief, SFN-ee were prepared at concentrations from 2.44 μg/mL to 2.50 mg/mL. Then, 90 μL of exponential-phase bacteria or fungal cells at 10^6^ CFU/mL were cultivated in a 96-well plate containing 10 μL of SFN-ee at various SFN concentrations. Plates were incubated for 24 h at 37 °C for bacterial strains and 30 °C for fungal strains. The absorbance value of each well was detected by a microplate reader (Thermo Fisher Scientific, Waltham, MA, USA). The lowest concentration without microorganism growth was determined as the MIC. In addition, the morphology of pathogens including *X. campestris*, *F. oxysporum*, *G. zeae*, *P. infestans*, and *S. aureus* treated with the SFN-ee at 2MIC and 4MIC concentrations were observed using an Eclipse 80i light microscope (Nikon, Tokyo, Japan).

### 2.8. Membrane Integrity Assay

To determine the membrane integrity of the microbial strains, exponential-phase bacteria or fungus cells (10^8^ CFU/mL) were transferred into Eppendorf tubes and treated with the SFN-ee at a dosage equivalent to 0.36 mg/mL SFN (dissolved in 4% DMSO, Cat:D8371, Solarbio, Beijing, China) in vitro for 2 h. The SFN-ee were removed by centrifugation. Then, the propidium iodide reagent (PI, 5 mg/mL, CAS:25535-16-4, Dingguo, Tianjin, China) was added to the tubes for incubation with the microbial strains, following the protocol described by Clementi et al. [28] with minor modifications. The microbial cells were washed with 1 mL of PBS (pH 7.4), then centrifuged, followed by the addition of 1 mL of PI reagent for 10 min. The PI reagent was then removed and the cells washed twice with PBS to remove the remaining dye. The membrane integrity of those bacteria or fungus cells was observed using a Zeiss Axio Imager Z1 fluorescence microscope (Zeiss, Oberkochen, Germany). The cells treated with 4% DMSO were used as a control.

### 2.9. Measurement of ROS Accumulation in Broccoli Leaves

The leaves of 30-day-old broccoli growing in the soil were sprayed with the SFN-ee at a dosage equivalent to 0.36 mg/mL SFN dissolved in 4% DMSO for 2 h. Leaves of 30-day-old broccoli were sprayed with 4% DMSO as a negative control, and leaves of 30-day-old broccoli without any treatment were used as a blank control. Subsequently, the leaves were soaked in 2.5 mg/mL diaminobenzidine (DAB, CAS:91-95-2, Sigma-Aldrich, Shanghai, China) for 12 h and decolored for 10 min in a solution of lactic acid/glycerin/ethanol (1/1/4, *V*/*V*/*V*, Guangshunda, Tianjin, China) to detect the level of H_2_O_2_ production. Similarly, the leaves of broccoli seedlings were harvested and treated with 0.5 mg/mL nitro blue tetrazolium (NBT, CAS:298-83-9, Sigma-Aldrich, Shanghai, China) for 12 h to assess the presence of O_2_^·−^. Production of O_2_^−^ and H_2_O_2_ content were then quantified according to the standardized method of Rai et al. [29]. In addition, the malondialdehyde (MDA) content of the leaves was determined according to the standardized method described by Wu et al. [30]. The expression levels of genes associated with ROS production were analyzed by qRT-PCR using the specific primers (Table S3).

### 2.10. Statistical Analysis

All statistical analyses were performed using GraphPad Prism 9 software (Graph-Pad Software Inc., San Diego, CA, USA). Data are represented as the mean ± standard deviation (SD). A paired sample *t*-test was used to compare the two groups, typically involving a control group and an experimental group.

## 3. Results

### 3.1. Molecular IDs of 33 B. oleracea Varieties Were Identified

**Figure 1:**
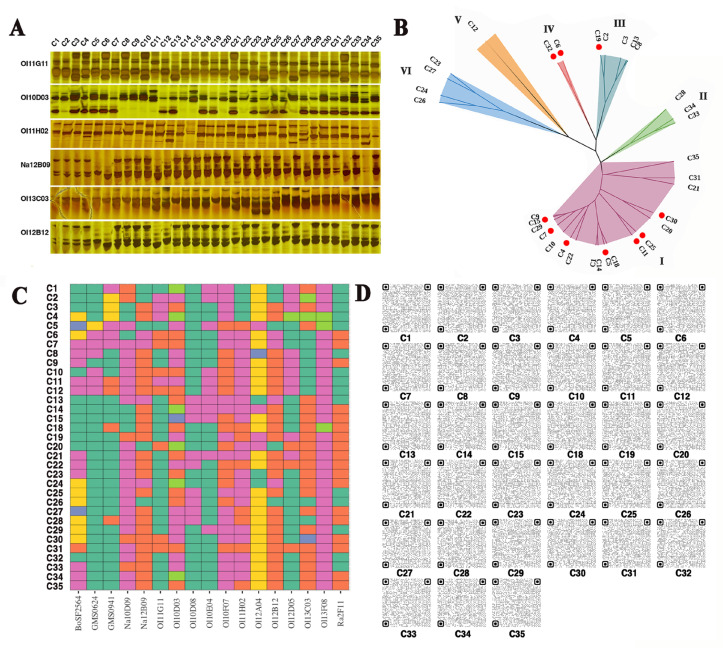
Representative DNA polymorphic fragments detected by SSR assay (**A**), phylogenetic tree (**B**), heatmap (**C**), and fingerprint quick response (QR) code (**D**) of 33 *B. oleracea* varieties. “OI11G11”, “Ol10D03”, “Ol11H02”, “Na12B09”, “Ol13C03”, and “Ol12B12” indicate the representative SSR primers. The sequences of these primers are shown in Table S2. C1–15, C18–22, C25, and C28–35 indicate the 29 broccoli cultivars. C23–24 and C26–27 indicate the four cauliflower cultivars. The circles (**B**) represent the varieties with the top 10 GSL content out of 33 varieties (C15, C7, C4, C5, C11, C25, C30, C19, C6, C32).

To uncover the possible relationships among the GSL content, SFN content, and the genetic diversity of different broccoli cultivars, and to screen the broccoli cultivar or new breeding materials with high SFN content, four cauliflower varieties were used as controls. The genetic diversity of 29 broccoli cultivars was explored by the SSR method. Among these 33 cultivars, 17 out of 39 SSR primer pairs exhibited polymorphic amplification (Figure 1). In total, 42 alleles produced by these 17 primer pairs were used to construct a dendrogram of the 33 cultivars. According to the dendrogram, the 33 cultivars were divided into 6 major groups. Consistent with the expectations, the broccoli cultivars were divided into 5 genetically closely related groups (groups I–V), whereas the cauliflower cultivars were classed into an independent group (Figure 1B). However, the number of broccoli cultivars in each group was irregular. Over 60% of the broccoli cultivars (18 out of 29) belonged to group I, while group IV comprised only two cultivars. These results indicated that although the 29 broccoli cultivars originated from different breeding companies, most of them showed highly similar genetic diversity, implying that the genetic background of broccoli germplasms was relatively simple. Nevertheless, the 33 *B. oleracea* varieties were distinguished by the SSR assay. Based on different combinations of representative SSR molecular markers, the molecular ID of each variety, even those with very similar genetic diversity, was established (Figure 1C,D). The results provided solid genetic data to evaluate the GSLs or SFN content of different broccoli cultivars.

### 3.2. Genetic Background of Different B. oleracea Varieties Influences GSL Content

**Figure 2:**
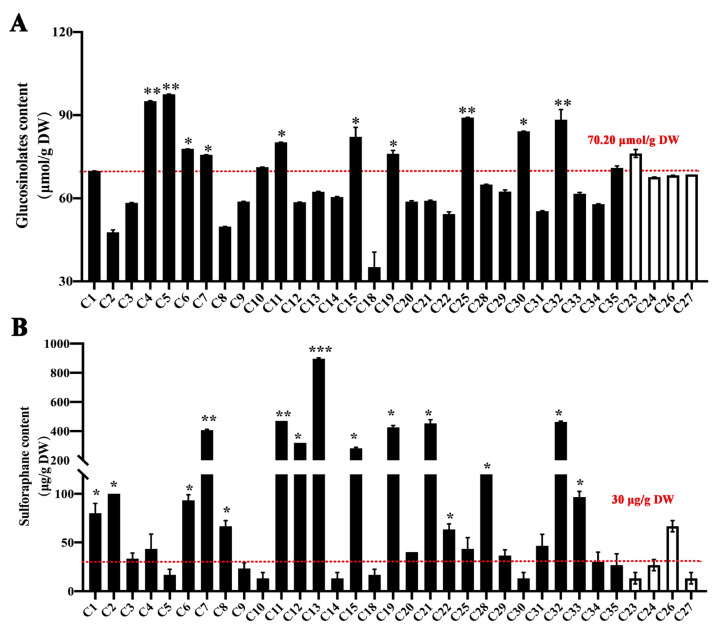
Content of GSLs (**A**) and SFN (**B**) in 33 *B. oleracea* varieties. The number highlighted in red represents the average content of GSLs and SFN in 4 cauliflower cultivars. C1–15, C18–22, C25 and C28–35 indicate the 29 broccoli cultivars. C23–24 and C26–27 indicate the 4 cauliflower cultivars. Data are based on three independent biological replicates (means ± SD, *n* = 15; Student’s *t*-test; * *p* < 0.05, ** *p* < 0.01, *** *p* < 0.001).

The relationship between the genetic background and GSLs content in 33 *B. oleracea* varieties was evaluated. The data indicated that four cauliflower cultivars had similar GSL content ranging from 67.66 μmol/g DW to 76.20 μmol/g DW, with the average GSL content being 70.20 μmol/g DW (Figure 2A). Similarly, although GSL content showed a large fluctuation (ranging from 35.16 μmol/g DW to 97.54 μmol/g DW) among the 29 broccoli cultivars, those classed into the same genetic group generally had similar GSL content (Figure 2A). Moreover, 10 out of the 29 broccoli cultivars holding a significantly higher GSL content than the average GSL content of four cauliflower cultivars were predominantly distributed in groups I and IV (Figure 1B and Figure 2A). These results confirmed that the GSL content was influenced by the genetic background of the different *B. oleracea* varieties. GSL content could be assessed based on the molecular IDs established for the 33 *B. oleracea* varieties, which offered a fast and simple strategy to estimate the GSL content before performing the extraction operation.

In addition to GSL content, SFN, the hydrolysate of GSLs, was also detected. Consistent with previous reports [31], the cauliflower cultivars displayed relatively lower SFN content than most of the broccoli cultivars, which could explain why broccoli, but not cauliflower, is the preferred material for SFN extraction in commercial production. Among the 29 broccoli cultivars, the SFN content of 15 broccoli cultivars was significantly higher than the average of the four cauliflower cultivars (30 μg/g DW) (Figure 2B and Figure S1). However, the 15 broccoli cultivars with a high SFN content did not fully correspond to those with a high GSL content (Figure 2A,B). Seven out of the fifteen broccoli cultivars, such as C2, C8, C12, C21, C22, C28, and C33, produced high SFN, but their GSL content were not particularly remarkable (Figure 2A,B). Moreover, different from GSL content, SFN content showed greater fluctuations among the 33 *B. oleracea* varieties (Figure 2B). The SFN content of C13 was about 67.25 times higher than that of C23 and C27 (Figure 2B and Figure S1). These results suggested that although SFN is the hydrolysate of GSLs, GSLs are essential but not the only factor affecting the SFN production. The correlation between the SFN content and genetic background of these varieties was more complex. Nevertheless, the molecular IDs established for the 33 *B. oleracea* varieties can distinguish these cultivars with high SFN content. This provided valuable information for screening and evaluating whether the materials used in SFN extraction truly have a high SFN biosynthetic capacity. Based on the molecular IDs and SFN content assay, the broccoli cultivar, C13, bred by our team has been demonstrated to hold high SFN accumulation, and was used as the raw material for SFN extraction in the subsequent assay.

### 3.3. Myrosinase and ESP Genes Play Important Roles in Affecting the SFN Content besides Genetic Background

**Figure 3:**
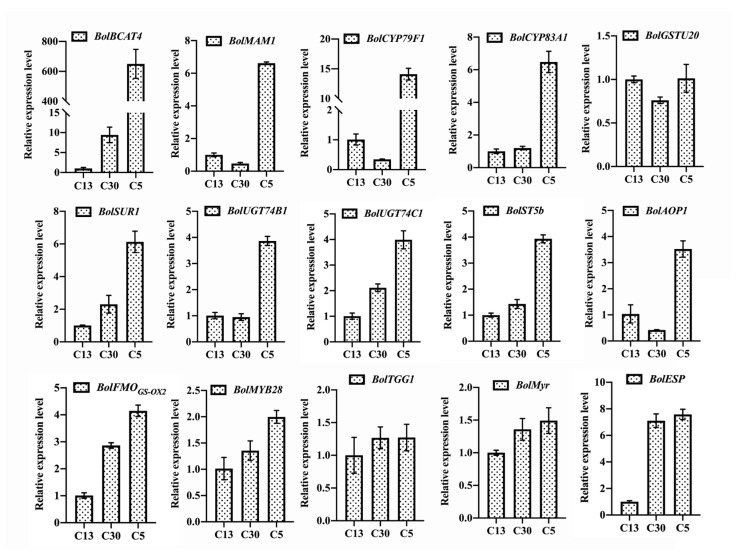
Quantitative expression levels of genes involved in GSL biosynthesis detected by qRT-PCR assay in three representative broccoli cultivars (C13, C30, C5). Mean values were from three independent technical replicates and normalized using *BolActin* transcripts, the error bars indicate the SD values (*n* = 15). The experiment was repeated twice with similar results.

In addition to the genetic background uncovered by SSR assay, the transcriptional expression levels of genes involved in GSL biosynthesis and SFN production were explored in three representative broccoli cultivars, namely, C13, C30, and C5 (Figure 3). The GSL content of these three cultivars was relatively high and showed a gradual uptrend. In addition, C13 holds the highest SFN content among the 33 *B. oleracea* varieties, while the SFN content of C30 and C5 was very low (Figure 2B). Different from the decreasing trend of SFN content, among the 12 representative genes involved in positively regulating GSL biosynthesis, the expression levels of almost all of the genes (11 out of 12), such as *BolBCAT4*, *BolCYP83A1*, *BolSUR1*, *BolUGT74C1*, *BolST5b*, *BolFMO_GS-OX2_*, and *BolMYB28*, exhibited an uptrend (Figure 3), corresponding to the uptrend of GSL content (Figure 2A). This result further confirmed that the genetic regulators play crucial roles in GSL accumulation. However, as mentioned above, SFN content was not directly correlated with GSL content and the expression levels of genes regulating GSL biosynthesis. Consequently, to further uncover the possible genetic factors affecting SFN production, the transcriptional expression levels of three key genes participating in regulating SFN production by hydrolyzing GSLs, namely, *BolTGG1*, *BolMyr*, and *BolESP*, were analyzed (Figure 3). The results demonstrated that while the expression levels of *BolTGG1* and *BolMyr*, both facilitating SFN production, were relatively high, they did not differ remarkably in C13, C30, and C5, even though the SFN content of C13 was considerably higher than that in C30 and C5 (Figure 2B and Figure 3). Interestingly, the expression level of *BolESP*, which is harmful to SFN production, in C30 and C5 cultivars with low SFN content was six times higher than that in C13, which had the highest SFN content (Figure 2B and Figure 3). These findings indicated that although myrosinase played important roles in SFN production, high ESP enzymatic activity was obviously unfavorable for SFN accumulation.

### 3.4. SFN Is Efficiently Extracted from Broccoli Curds

SFN is mostly extracted from the seeds or seedlings of broccoli; however, the seed yield of broccoli germplasms is very low. Low seed yield is a huge bottleneck that greatly increases the cost of SFN extraction. In the present study, to reduce the cost of SFN extraction due to the expensive broccoli seeds, the broccoli curds with large biomass were used to extract SFN. Subsequently, SFN was extracted from the curds of the broccoli cultivar, C13, which had the highest SFN content among the tested cultivars (Figure 2B). The data indicated that the SFN content extracted from curds and seedlings of C13 was 628.44 μg/g DW and 896.67 μg/g DW, respectively. The extracted SFN yield from the curds was about 70% of that from the seedlings. Nevertheless, in consideration of the obviously higher biomass and lower cost of curds compared to seedlings, these results confirmed that the extraction method established here could also efficiently extract high SFN from broccoli curds and was more feasible for the large-scale extraction of SFN.

### 3.5. SFN-ee Display Strong and Broad-Spectrum Antimicrobial Activity In Vitro

**Figure 4:**
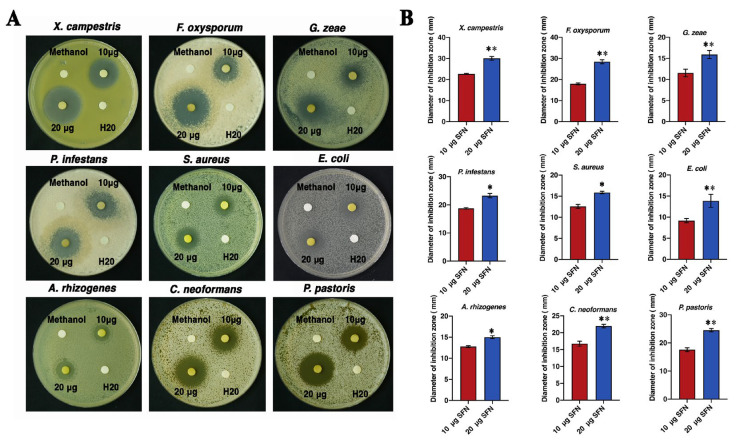
Antimicrobial activity (**A**) and inhibition zone diameter (**B**) of SFN-ee against 9 tested microorganisms in vitro. 10 μg and 20 μg indicated the disks loading the SFN-ee in the dosages equivalent to 10 μg and 20 μg SFN, respectively. The disks containing 10 or 20 μg SFN were then placed on the petri dishes with different microbial strains. Methanol was used as the negative control and sterile water (H_2_O) was used as the blank control. Data are based on three independent technical replicates (means ± SD; * *p* < 0.05, ** *p* < 0.01; Student’s *t*-test; *n* = 6).

The antimicrobial activity of SFN-ee from broccoli curds was evaluated against 9 microorganisms, including 4 plant pathogens and 1 human pathogen (Figure 4A). The data confirmed that the inhibition zone diameters of SFN-ee with 10 μg SFN ranged from 9.16 to 22.68 mm against the 9 microorganisms (Figure 4B). As the dose increased, SFN-ee containing 20 μg SFN produced larger inhibition zones, with diameters ranging from 13.86 to 30.06 mm, compared to those produced by SFN-ee containing 10 μg SFN. In particular, the inhibition zones of SFN-ee with 20 μg SFN upgraded to 30.06, 28.45, 15.90, 23.25, and 15.86 mm against the plant pathogens *X. campestris*, *F. oxysporum*, *G. zeae*, and *P. infestans*, and the human pathogen, *S. aureus*, respectively (Figure 4B). The results confirmed that SFN-ee displayed antimicrobial activity in vitro against the tested microorganisms, especially against the four plant pathogens, and the antimicrobial activity of SFN-ee was dose dependent.

**Figure 5:**
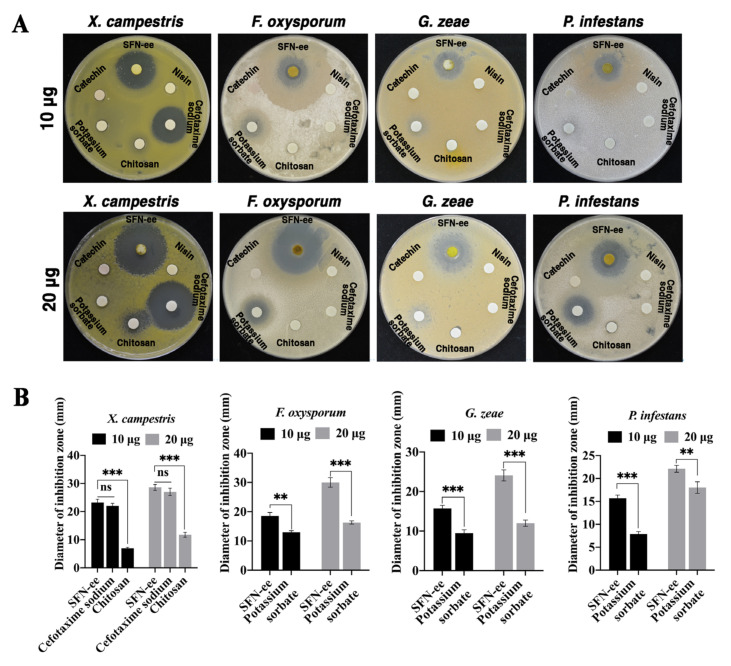
Comparison of antimicrobial activity (**A**) and inhibition zone diameters (**B**) of SFN-ee and 5 representative antimicrobial agents against 4 plants pathogens in vitro. 10 μg and 20 μg indicated the content of antimicrobial agents carried on the disks, respectively. Five representative antimicrobial agents were used as positive controls. Antimicrobial agents that did not produce inhibitory activity against plant pathogens did not analyze the diameter of inhibition zone. Data are based on three independent technical replicates (means ± SD; ** *p* < 0.01; *** *p* < 0.001, Student’s *t*-test; *n* = 6).

The antimicrobial effect of SFN-ee was then compared with five representative antimicrobial agents: nisin, cefotaxime sodium, chitosan, potassium sorbate, and catechin (Figure 5). The data showed that SFN-ee, cefotaxime sodium, and chitosan all inhibit the bacterial pathogen *X. campestris*, with their antibacterial activities being dose dependent. In contrast, nisin, potassium sorbate, and catechin did not exhibit antibacterial activity against *X. campestris* at content of 10 μg and 20 μg (Figure 5A). By comparing the diameters of inhibition zones in *X. campestris*, it was found that at both dose levels, the diameters of SFN-ee’s inhibition zones (23.21 mm, 28.60 mm) were similar to those of cefotaxime sodium (22.03 mm, 27.02 mm), but both were significantly larger than those of chitosan (6.97 mm, 11.77 mm) (Figure 5B). This result indicated that SFN-ee exhibited strong antibacterial activity, and its antibacterial activity was comparable to that of antibiotics. For three plant fungal pathogens (*F. oxysporum*, *G. zeae*, and *P. infestans*), SFN-ee and potassium sorbate exhibited antifungal activity (Figure 5A). The diameters of SFN-ee’s inhibition zones against the three plant fungal pathogens were considerably larger than those of potassium sorbate at both tested dosages. This comparison suggested that the antifungal activity of SFN-ee was significantly stronger than that of potassium sorbate (Figure 5B). However, nisin, cefotaxime sodium, chitosan, and catechin did not have antifungal activity against three fungal pathogens at contents of 10 μg and 20 μg. Therefore, the results confirmed that SFN-ee exhibited broad-spectrum and strong antimicrobial activity compared to other well-known antimicrobial agents, and had great potential for applications in the field of disease resistance, particularly against plant fungal pathogens.

### 3.6. SFN-ee Inhibit the Proliferation and Destroy the Membrane Integrity of Pathogens

**Figure 6:**
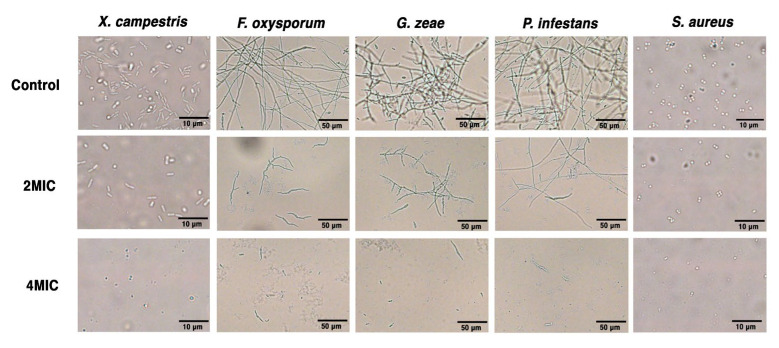
Morphological characters of 5 pathogens treated by SFN-ee under optical microscope. The pathogens were treated by SFN-ee in the SFN dosages equivalent to 2MIC and 4MIC for 24 h, respectively. For *X. campestris* and *S. aureus*, scale = 10 µm. For *F. oxysporum*, *G. zeae*, and *P. infestans*, scale = 50 µm.

**Figure 7:**
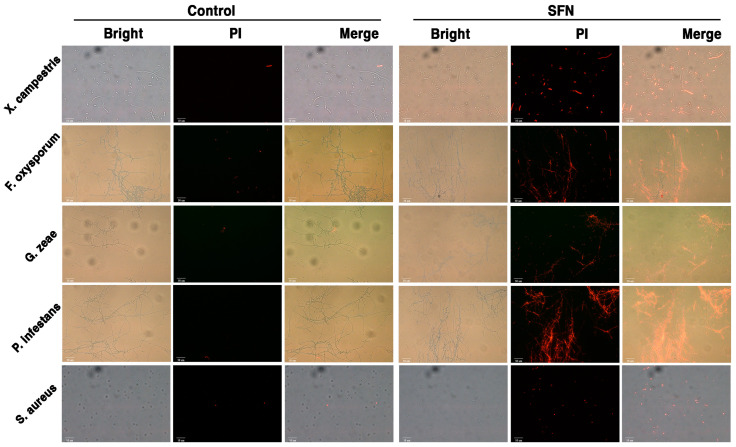
Membrane integrity of 5 pathogens treated by SFN-ee. The pathogens were treated by SFN-ee in a dosage equivalent to 0.36 mg/mL SFN for 2 h. The cells treated with 4% DMSO were used as a control. The cells of these pathogens were then stained with 5 mg/mL PI. Red fluorescent signal indicated the membranes of pathogenic cells were permeabilized. For *X. campestris* and *S. aureus*, scale = 10 µm. For *F. oxysporum*, *G. zeae*, and *P. infestans*, scale = 50 µm.

To further understand the antimicrobial activity and mechanism of SFN-ee, the MICs of SFN-ee against *X. campestris*, *F. oxysporum*, *G. zeae*, *P. infestans*, and *S. aureus* were determined. The results confirmed that the MICs were 15.63 μg/mL for *X. campestris*, *F. oxysporum*, and *P. infestans*, 7.81 μg/mL for *G. zeae*, and 31.25 μg/mL for *S. aureus* (Table S4). Subsequently, the morphologies of the five pathogens, including 4 plant pathogens and 1 human pathogen, treated with SFN-ee containing the SFN at concentrations of 2MIC and 4MIC, were characterized in vitro (Figure 6 and Figure 7). The proliferation of *X. campestris* and *S. aureus*, two pathogenic bacteria harming plants and humans, respectively, was significantly inhibited by the SFN-ee at 2MIC. Consistent with the dose-dependent antimicrobial activity of SFN-ee, the cell numbers of the two bacteria continuously decreased as the concentration of SFN-ee increased to 4MIC (Figure 6). Moreover, the morphology of the survival *X. campestris* cells was uneven and some of them had obvious partial collapse and tortuosity, compared to the untreated control. Similarly, under the treatment of the SFN-ee with 2MIC and 4MIC, the proliferation of the other three tested plant pathogens, which all belong to fungi, showed dose-dependent inhibition. The SFN-ee at 4MIC could almost entirely inhibit the growth of mycelia, with only a few sporadic spores surviving (Figure 6). Furthermore, the PI staining assay indicated that the five pathogens treated with SFN-ee displayed stronger fluorescence intensities than those of the untreated controls (Figure 7). Stronger fluorescence intensities mean more serious cell membrane damage. Consequently, the results suggested that destroying the integrity of pathogenic cell membrane is one of the important functions for SFN-ee to inhibit the proliferation of pathogens. These findings suggested that SFN as well as its analogues play an antimicrobial role in part by disrupting the integrity of pathogen membranes.

### 3.7. SFN-ee Induce Endogenous ROS Accumulation in Broccoli Leaves

**Figure 8:**
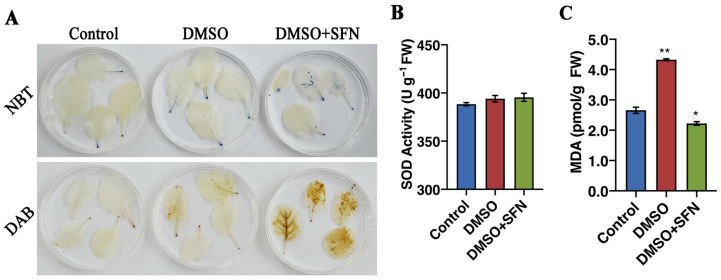
Physiological characteristics assay of broccoli leaves treated by SFN-ee. (**A**) H_2_O_2_ and O_2_^·−^ detection in broccoli leaves treated by SFN-ee using 0.5 mg/mL NBT and 2.5 mg/mL DAB staining, respectively. (**B**,**C**) SOD activity and MDA content in broccoli leaves treated by SFN-ee, respectively. The error bars in (**B**,**C**) indicate the SD values. Asterisks indicate significant differences (* *p* < 0.05, ** *p* < 0.01; Student’s *t*-test; *n* = 6).

In addition to directly inhibiting the proliferation of pathogens, the role of SFN, as an endogenous hydrolysate, in regulating plant defense response was explored. The leaves of broccoli seedlings were then treated with SFN-ee in vitro, and the ROS accumulation was observed. The results confirmed that the ROS production system was immediately triggered by the exogenous SFN-ee. More ROS accumulated in leaves treated with SFN-ee than that in the untreated control (Figure 8A), but no remarkable difference was observed in the activity of SOD, which functions to scavenge ROS (Figure 8B).

**Figure 9:**
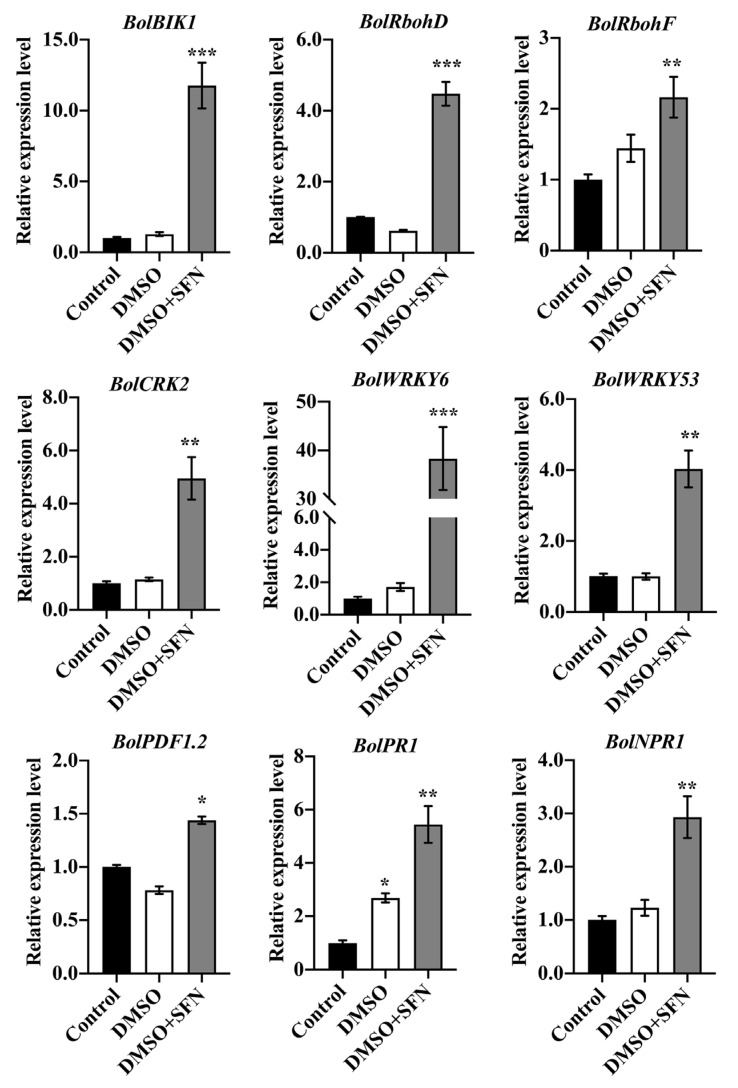
Quantitative expression levels of genes involved in ROS generation and pathogen defense in leaves treated by SFN-ee. Control represented a blank control without applying any treatment to 30-day-old broccoli leaves, DMSO represented a negative control of 4%DMSO applied to 30-day-old broccoli leaves, and 30-day-old broccoli leaves were sprayed with 4% DMSO-dissolved SFN as an experimental group. Mean values were from three independent technical replicates and normalized using *BolActin* transcripts, the error bars indicate the SD values (Student’s *t*-test, *n* = 15; * *p* < 0.05, ** *p* < 0.01, *** *p* < 0.001). The experiment was repeated twice with similar results.

Consistent with the ROS accumulation, the expression levels of marker genes involved in ROS production, such as Botrytis-Induced Kinase 1 (*BIK1*), Respiratory Burst Oxidase Homolog D (*RbohD*), Respiratory Burst Oxidase Homolog F (*RbohF*), and Cysteine-Rich Rlk2 (*CRK2*) were sharply increased in the leaves treated with SFN-ee (Figure 9). In addition, the data indicated that the MDA content in the broccoli leaves treated with SFN-ee did not increase compared with the untreated control (Figure 8C), suggesting that the leaves did not suffer from membrane lipid oxidation damage under the treatment with SFN-ee. The results indicated that the application of exogenous SFN-ee induced ROS accumulation. Moreover, qRT-PCR assay further confirmed that the defense-related genes, such as Non-expresser of Pathogenesis Related 1(*NPR1*), *WRKY6*, *WRKY53*, Pathogenesis Related 1 (*PR1*), and Plant Defensin 1.2 (*PDF 1.2*), displayed higher expression levels in leaves treated with SFN-ee than that in the untreated control (Figure 9). These findings suggested that SFN-ee can function as endogenous compounds in the ROS-mediated signaling pathway to defend against pathogenic infection in plants.

## 4. Discussion

SFN is a promising phytochemical and has caught the attention of the scientific community due to their chemopreventive properties, extensive anticancer effects, and antibacterial activity [32]. SFN and its precursors, GSLs, widely exist in broccoli, cauliflower, Brussels sprout, and cabbage, which are all classified as *B. oleracea* species. However, the SFN and GSL content of these vegetables are obviously different [33,34,35]. Previous investigations indicated that same vegetables but with different cultivars have uneven SFN and GSL content partly because of their different genetic backgrounds [36,37,38,39]. In this study, the phylogenetic tree combined with GSL contents analysis also implied that the GSL contents of different broccoli cultivars were closely related to their genetic backgrounds. Broccoli cultivars with high GSL contents often had similar genetic backgrounds. Moreover, the gene expression profile assay indicated that the genes involved in regulating GSL biosynthesis, such as *BolBCAT4*, *BolMYB28*, *BolUGT74B1*, *BolUGT74C1*, and *BolCYP83A1*, displayed higher transcriptional expression levels in broccoli cultivars with a high GSL content than in those with low GSL content. Investigations have demonstrated that all these genes are involved in positively regulating the biosynthesis of GSLs [40,41,42]. For example, overexpression of *BnaC2.MYB28* leads to extremely high GSL content in *B. napus* seeds, whereas *BnaC2.MYB28* knockout results in reduced GSL content [42]. The absence of *UGT74B1* shows a decrease in GSL content in *Arabidopsis*. By contrast, overexpression of *UGT74C1* in *ugt74b1* plants largely alleviates the low-GSL chemotype [41]. These pieces of evidence confirmed that the genetic regulation plays a crucial role in GSL content. However, compared with the close correlation between the genetic factors and GSL content, the internal factors affecting SFN content are more ambiguous. Nevertheless, the results indicated that the SFN contents are affected not only directly by the GSL contents, but also by the enzymatic activity of myrosinase and ESP in broccoli. *BolTGG1* and *BolMyr* are genes coding myrosinase, which functions in directly hydrolyzing glucoraphanin to SFN [9,43]. Unexpectedly, compared to broccoli cultivars with a low SFN content, the expression levels of both genes were not significantly increased in cultivars with high SFN content. Inversely, the expression level of *ESP* significantly decreased in broccoli cultivars with high SFN content. ESP hydrolyzes glucoraphanin to nitrile but not SFN in the GSL metabolic pathway [10,35]. Decreased ESP activity can lead to increased SFN formation in broccoli [44]. Consequently, *ESP* negatively regulates SFN production in broccoli. These findings indicated that besides genetic background and GSL content, ESP activity is a crucial factor that determine SFN production in broccoli. Broccoli cultivars with strong GSL biosynthetic ability, high myrosinase, and low ESP enzymatic activity are the preferred materials for SFN production.

Over the last few decades, the anticancer effects of SFN against different types of cancer, including breast, ovarian, prostate, colon, lung, and gastric cancer have been widely reported by different studies in vivo and in vitro [45,46,47,48,49]. SFN, acting as an antioxidant, can inhibit the activity of mutagenic factors (phase I) and activate phase II enzymes involved in the detoxification of carcinogens. This action helps prevent DNA damage, inhibit tumor initiation, restrict cancer cell proliferation, and limit mutated cancer cell metastasis [50,51]. However, the antibacterial properties of SFN are not as widely explored as their anticancer activity. The antimicrobial effects and mechanism of SFN against diverse pathogens, especially plant pathogens, remain ambiguous. This result confirmed that SFN-ee inhibited the proliferation of nine microorganisms, including bacteria and fungi, suggesting that SFN-ee have broad-spectrum antimicrobial properties. Further comparisons with the five representative antimicrobial agents revealed that the SFN-ee displayed strong antimicrobial activity in vitro against four plant pathogens: *X. campestris*, *F. oxysporum*, *P*. *infestans*, and *G*. *zeae* (Figure 4 and Figure 5). The MIC values of SFN-ee against *X. campestris*, *F. oxysporum*, *P. infestans*, and *G. zeae* were 15.63 μg/mL, 15.63 μg/mL, 15.63 μg/mL, and 7.81 μg/mL, respectively, which were generally lower than those reported in the research of the five representative antimicrobials. Furthermore, previous investigations also indicated that the MIC values of these five well-known antimicrobial agents and other plant natural products were not lower than that of SFN-ee. For example, nisin is a natural preservative used in many food products. It can inhibit pathogenic foodborne bacteria and many other Gram-positive microorganisms. MIC value of nisin for *S. aureus* is 30 µg/mL [52]. Potassium sorbate is the antimicrobial food additive which has the great inhibitory effect on *L. lactis* growth, at a MIC of 25 mg/mL [53]. Chitosan has attracted a growing attention as a food preservative due to its versatility, nontoxicity, biodegradability, and biocompatibility, showing activity against *S. aureus* with an MIC of 390 µg/mL [54,55]. Catechins are polyphenolic compounds found in various plants such as green tea, which possess a bactericidal effect against black-pigmented, Gram-negative anaerobic rods (BPR) in vitro, with an MIC of 1.0 mg/mL [56]. The antibacterial effect of ceftazidime against 41 clinical *S. maltophilia* isolates is also evaluated. The results demonstrated that MIC50 values for ceftazidime were 64 μg/mL [57]. In addition, allyl isothiocyanate (AITC) is a kind of bioactive molecule with antibacterial properties, demonstrating antibacterial activity against *E. coli*, *P. aeruginosa*, *S. aureus*, and *L. monocytogenes*. The MIC of AITC for all tested bacteria was 100 μg/mL [58]. The MIC of phenethyl isothiocyanate (PEITC), which is also prevalent in cruciferous plants, ranges from 41 to 81 μg/mL [15]. Flavonic acid BB, derived from plants, inhibits clinical *S. haemolyticus* strains and their biofilms. The MIC of flavanolic acid BB against 16 clinical strains of *S. haemolyticus* ranged from 5 to 480 µg/mL [59]. The extracts of *S. scordifolia* containing flavones, luteolin, and apigenin showed activity against *Malassezia furfur*, with GMMIC50 values of 64 mg/mL [60]. These findings indicated that compared with other natural products, preservative and even some antibiotics, the SFN-ee obtained in the present study exhibited a better antimicrobial activity, which suggested the great potential of SFN-ee as biological control agent against plant pathogens.

SFN is commonly released as a defensive component when plants suffer from pathogen infection, insect attack and other biotic or abiotic stresses [9]. A few investigations have been conducted to uncover the mechanism of SFN in defending against pathogen infection. Nowicki et al. [61] demonstrated that SFN can inhibit the growth and proliferation of several clinical pathogen isolates, such as *E. coli*, *B. subtilis*, and *E. faecalis*, by destroying the membrane integrity of these pathogens. Similarly, moringin, an analogue of SFN from *Moringa oleifera* seeds, displays strong antimicrobial activity against *L. monocytogenes*, significantly increasing the permeability of the cell wall and cell membrane [62]. Consistent with previous reports, the present results identified that the membrane integrity of both plant and clinical pathogens was also destroyed by SFN-ee. These findings indicated that directly destroying the membrane integrity of pathogens is one of the important roles of SFN in exerting antibacterial effect. In addition, as an endogenous hydrolysate in plants, besides exerting antibacterial activity by destroying the membrane integrity of external pathogens, it remains unclear whether SFN could act on the plant itself. The present results revealed that exogenous application of SFN-ee could induce ROS accumulation and increase the expression levels of defense-related genes in broccoli leaves. ROS production constitutes a crucial defense response in plants for recognizing pathogen infections and serves as a signal that activates plant defenses against biotic stresses. This process facilitates the initiation of various defensive actions by regulating the expression of genes related to defense mechanisms [63]. One of the quickest defense reactions to pathogen attack is to produce ROS at the site of attempted invasion, namely oxidative burst in plants [64]. ROS bursts at the infection sites can trigger a hypersensitive response, which lead to rapid programmed cell death in a few distinct cells. This process can impede the invasion of pathogens because they need to obtain nutrients from living cells [65]. Moreover, ROS could directly inhibit the growth of pathogens or kill the pathogens, especially in the case of more reactive species like hydroxyl radicals [66]. These pieces of evidence indicated that in addition to directly inhibiting or killing pathogens, activating an ROS-mediated defense reaction could be another crucial role for SFN to defending against pathogen infection in plants.

## 5. Conclusions

In summary, the genetic factors that could evaluate the GSL content and SFN biosynthetic ability among 33 different *B. oleracea* varieties were identified. A low-cost method utilizing broccoli curds but not seeds or seedlings as raw materials to extract SFN was established, yielding extracts with high SFN content. The SFN-ee were confirmed to exhibit strong antimicrobial activity against multiple plant pathogens. Subsequently, the antimicrobial mechanism of the SFN-ee was explored. The results indicated that SFN-ee play a dual role in defending against pathogens by directly inhibiting pathogenic cells and activating the ROS signaling pathway in plants. These findings provided new insights into the function and mechanism of SFN against pathogens, and suggested the potential application of SFN-ee as natural biocontrol agent in protecting crops from pathogenic infection and in food preservation.

## Data Availability

Data are contained within the article.

## Supplementary Materials

The following supporting information can be downloaded at: https://www.mdpi.com/article/10.3390/biom14030352/s1, Figure S1: SFN content in 33 *B. oleracea* varieties detected by HPLC assay; Table S1: 33 *B. oleracea* varieties and DNA polymorphisms detected by SSR assay; Table S2: Primers used in SSR analysis; Table S3: Primers used in qRT–PCR assay; Table S4: MIC of SFN-ee against 5 pathogens.
