# Supplementary material for: Sulforaphane-Enriched Extracts from Broccoli Exhibit Antimicrobial Activity against Plant Pathogens, Promising a Natural Antimicrobial Agent for Crop Protection
Source: Biomolecules. 2024 Mar 14;14(3):352. doi: 10.3390/biom14030352 (PMC10968597; doi:10.3390/biom14030352)
Supplement: Supplementary file 1 [file biomolecules-14-00352-s001.zip › Supplementary Figure S1.pdf]

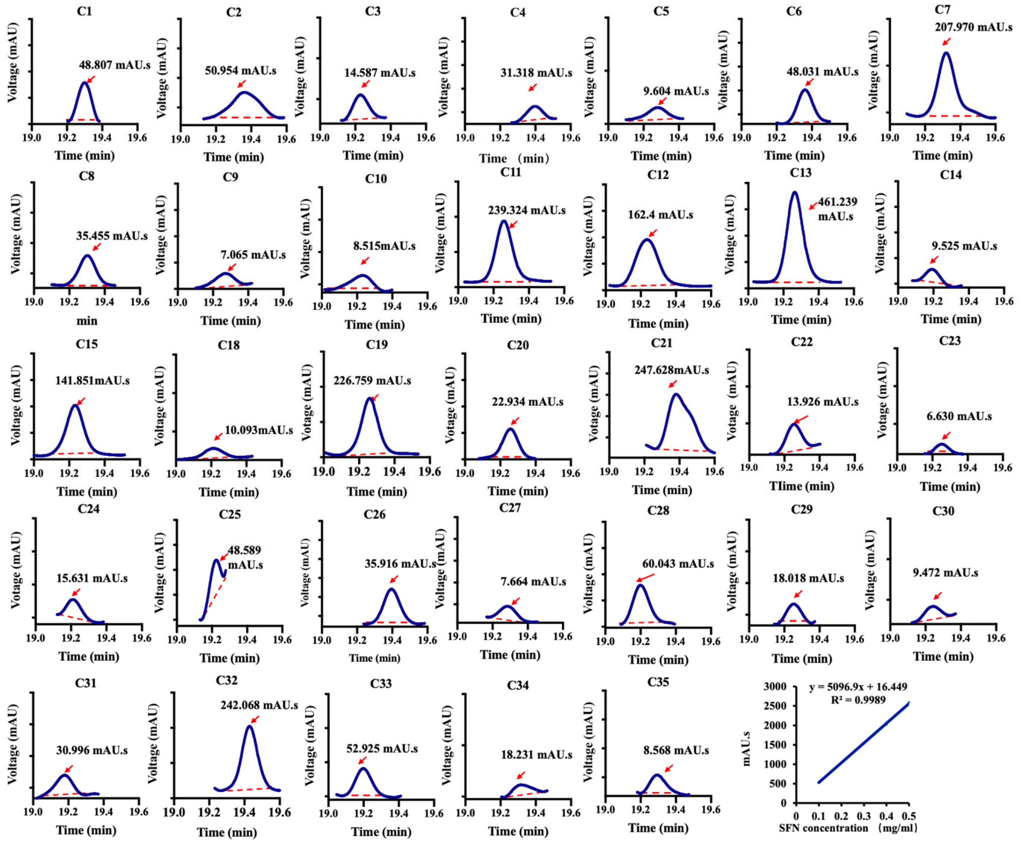

Figure S1 SFN content in 33 *B. oleracea* varieties detected by HPLC assay. The numbers following the red arrows indicate the peak area, which is positively correlated with SFN content. The correlation of SFN concentration and peak area is showed in the bottom right of the figure (R^2=0.9989). C1-15, C18-22, C25 and C28-35 indicate the 29 broccoli cultivars. C23-24 and C26-27 indicate the 4 cauliflower cultivars.
